# Moral distress in psychiatric nurses in Covid-19 crisis

**DOI:** 10.1186/s40359-023-01048-y

**Published:** 2023-02-17

**Authors:** Nahid Tavakol, Zahra Molazem, Mahnaz Rakhshan, Omid Asemani, Shahpar Bagheri

**Affiliations:** 1 Department of Nursing, School of Nursing and Midwifery, Community Based Psychiatric Care Research Center, Shiraz University of Medical Science, Shiraz, Islamic Republic of Iran; 2 Department of Nursing, School of Nursing and Midwifery, Shiraz University of Medical Sciences, Zand St., Namazee Sq., Shiraz, 7193613119 Islamic Republic of Iran; 3 Community Based Psychiatric Care Research Center, School of Nursing and Midwifery, Shiraz University of Medical Sciences, Shiraz, Islamic Republic of Iran; 4 Department of Medical Ethics and Philosophy of Health, Faculty of Medicine, Shiraz University of Medical Sciences, Shiraz, Islamic Republic of Iran; 5 Center for Interdisciplinary Research in Islamic Education and Health Sciences, Shiraz University of Medical Sciences, Shiraz, Islamic Republic of Iran; 6 Department of Nursing, School of Nursing and Midwifery, Community Based Psychiatric Care Research Center, Shiraz University of Medical Sciences, Shiraz, Islamic Republic of Iran

**Keywords:** Psychiatric, Nurse, Moral, COVID-19, Iran

## Abstract

**Background:**

The COVID-19 pandemic has put heavy pressure on nurses. Psychiatric nurses are also exposed to moral distress due to the special conditions of psychiatric patients and patient’s lack of cooperation in observing health protocols. This study has been conducted to explore and describe factors that caused moral distress in Iranian psychiatric nurses during the COVID-19 pandemic.

**Method:**

This qualitative study with a conventional content analysis approach involved 12 nurses at the Shiraz University of Medical Sciences in Iran. This study was conducted in the winter of 2021. Data collection was performed by semi-structured interviews, data analysis was performed based on the five steps of Graneheim and Lundman.

**Results:**

By continuous comparison and integration of data, 17 subcategories, 8 subcategories, and 3 categories were extracted from 252 initial codes. The causes of moral distress in psychiatric nurses during the COVID-19 pandemic were identified as emotional responses (Fear and Doubt), relational factors (Nurses’ Relationship with Each Other, Nurse-physician Relationship, and Relationship whit Patients), and Institutional factors (Lack of Attention to Health Instructions, Failure to complete the treatment process for patients and Institutional Policies).

**Conclusion:**

New dimensions of the causes of moral distress associated with the COVID- 19 pandemic are discovered in this study. Managers and planners should equip psychiatric hospitals with isolation facilities and Personal Protection Equipment for patients and nurses. Strengthening the ethical climate by improving communication skills and individual nursing empowerment to prevent moral distress is recommended.

**Supplementary Information:**

The online version contains supplementary material available at 10.1186/s40359-023-01048-y.

## Background

The COVID-19 outbreak, which occurred around 2020, caused many deaths worldwide [1]. In Iran, on February 19, 2020, the first case of this disease was reported, and Afterward, the disease spread very rapidly in all country [2]. Five waves from March 2019 to summer 2021 determined and caused 140,000 death so far in Iran [3].

Like other countries, the prevalence of this disease has led to many challenges for the Iranian healthcare system and nurses encounter ethical issues they usually do not experience every day [4]. Public health emergencies with shortages of resources can create moral distress and affect patient care and the safety of healthcare workers [5]. Nurses caring for COVID-19 patients reported high levels of occupational burnout, low levels of resilience, and high levels of tension [6]. Ethical issues particular to the psychiatric nurses in COVID pandemic may cause some to experience so-called “moral distress”.

Moral distress occurs when a person knows what is right but has prohibited it due to organizational obstacles. Jameton views moral distress as contrary to his moral values linked to internal or external barriers [7, 8]. They feel pressured to support patients and make the best ethical decisions, making them exhausted, powerless, and anxious. Helplessness, guilt, self-criticism, and low self-esteem can lead to physical symptoms of moral distress, including sleep disturbances, crying, or loss of appetite [9]. It also negatively affects patient care, leads to avoidance of certain clinical situations, and may cause quitting the nursing profession [10, 11].

Psychiatric patients are more susceptible to the disease of COVID compared to general patients [12]. Psychiatric inpatients usually take part in group activities to maintain their social functions. Moreover, patients in psychiatric hospitals are usually confined to crowded living environment. Because of their poor self-care ability and disordered mental state, they may not be able to take measures to protect themselves from infection [13]. All thing considered, psychiatric nurses are prone to be caught by COVID-19 due to a lack of proper isolation rooms and disregarding health protocols by psychiatric patients with insufficient insight into their health condition. As well as psychiatric nurses who work with psychotic patients in various stages of the disease [14] experience moral dilemmas and moral distress.

In a study in Iran, Hosseini Moghadam et al. [15] have reported that Iranian nurses have experienced occupational stress, anxiety and psychological tension during the outbreak of the Covid-19 pandemic, which has aggravated the creation of moral challenges and moral distress in nurses.

A study in China has reported that due to role conflict between family and work, conflict with the original professional value, inadequate preparedness for the outbreak, and higher exposure risks than peers in general hospitals, the stressors of psychiatric nurses has increased during the COVID pandemic[16].

Accordingly, due to the high possibility of psychiatric nurses being involved with the COVID-19 disease due to the condition of psychiatric patients and also the experience of moral issues and moral distress in this situation, it is necessary to investigate the causes of moral distress among psychiatric nurses influenced by COVID-19 pandemic.

Considering the above-mentioned points and the fact that the researchers could not find no studies which investigated causes of moral distress at in the COVID-19 pandemic, a qualitative study could obtain an in-depth understanding of the nurse’s experiences. Accordingly, the present study uses a qualitative approach to explore the causes and factors that create moral distress in psychiatric nurses who face and provide care to COVID-19 patients.

## Methods

### Study design and aim

In this study, a qualitative design using content analysis and individual semi-structured in-depth interviews was conducted until data saturation. Content analysis is an objective method of systematically describing and quantifying qualitative data and applied to classifications of generating initial codes, defining and naming themes, reviewing themes, and searching for themes. [17]. We tried to investigate the causes of moral distress in psychiatric nurses who cared for psychiatric patients in the COVID-19 crisis and their experiences.

### Participants and sampling

The sample population was 80 psychiatric nurses of Ibn Sina Psychiatric Hospital in Shiraz, Iran. Twelve psychiatric nurses in the winter of 2021 were selected by purposive sampling. Inclusion criteria included expressing feelings and experiences, having at least 6 months of work experience, and being interested in participating in the study. Exclusion criteria included not working in the inpatient wards and with patients.

### Data collection and data analysis

The interviews were recorded with the permission of the interviewees implemented verbatim, and analyzed immediately after the interview. Each interview duration was between 45 and 60 min. Each interview lasted from 40 to 60 min and began with a general question “Can you describe your experiences of a work shift in which you faced or cared for a COVID19 patient?” Subsequently, the interviewer asked more specific questions: “What are your experiences about the ethical issues when you faced and cared for COVID-19 patients?”, “What factors causes moral distress when face and care for COVID-19 patients?” Moreover, follow-up questions were asked to obtain more details about the objective of the study (Additional file 1: Interview Guide). The data analysis process was performed according to the proposed Granheim and Landman method [18] in five steps of (1) The recorded interviews were transcribed, (2) The researchers listened to the recordings and reviewed the transcripts several times to find the meaning units, (3) The meaning units from the statements of the participants were extracted in the form of initial codes, (4) Codes were categorized according to the conceptual similarities to be minimized, (5) This trend continued across all the analysis units until themes and subthemes emerged. Interviews continued until data saturation and when no new code was created in the last two interviews. The research team and two qualitative research experts examined the codes to verify that the data were saturated. The data were organized using MAXQDA 2010 distributed by VERBI.

### Analytic rigor

Trustworthiness, characterized as credibility, transferability, dependability and confirmability must be considered when assessing qualitative information [19]. To ensure credibility, two professional researchers were asked to examine and interpret the data, and all extracted codes and categories were verified and approved by the authors. To increase the acceptability and accuracy of the findings, the combination of several semi-structured interviews, re-checking the data with the interviewees, and simultaneous analysis of them by the researchers was utilized. Transferability was also operationalized by providing direct citations, rich modification of data, and consulting the professors in qualitative research. To develop the dependability of data, instant data transcription, sharing the colleagues’ opinions, and reviewing the manuscripts by the participants were done. The confirmability of data was guaranteed through consulting with some experts about the accuracy of the interpretations and the coding process (two faculty members of Shiraz School of Nursing and Midwifery and one faculty member of the Medical Ethics School). Moreover, the steps and methods of data extraction were accurately recorded.

## Results

Twelve nurses, including six female and six male nurses, were interviewed in this study. The mean age of nurses was reported as 31.5 ± 0.325 years. Also, 7 and 5 of the studied nurses were married and single. Their mean work experience was 8.8 ± 0.125 years, and 3 participants had managerial backgrounds (They were currently working in inpatient wards and cared for patients) (Table 1).

**Table 1:** demographic characteristics of participants

Demographic characteristics	count	%
Age		
20–29 years	5	41.66
30–39 years	5	41.66
40–49 years	2	16.66
Gender		
Male	6	50
Female	6	50
Marriage status		
Married	7	58.33
Single	5	41.66
Educational background (highest degree)		
BSC	10	83.33
MSC	2	16.66
Time employed		
1–5 years	5	41.66
6–10 years	2	16.66
11–15 years	3	25
16–20 years	1	8.33
21–25 years	1	8.33
Managerial backgrounds		
Yes	3	25
No	9	75

The primary analysis resulted in 252 initial codes, which finally led to 17 subcategories and eight categories. (Table 2).

**Table 2:** Categories, subcategories and sub subcategories

Categories	Subcategories	Sub subcategories
Emotional responses	Fear	Fear of COVID-19 disease and ineffective care of the patients by the nurse
		Fear of COVID-19 disease and inadequate treatment provided by physicians
	Doubt	Being doubtful in quitting the nursing profession in the Corona outbreak period
		Being doubtful in continuing nursing profession during Corona outbreak period
Relational factors	Nurses’ Relationship with Each Other	Working with careless colleagues
	Nurse-physician Relationship	Disregarding nurses' opinions in therapeutic decisions made by physicians
	Relationship whit Patients	Inadequate communication with the patient by nurses
		Insufficient communication with the patient by physicians
Institutional factors	Lack of Attention to Health Instructions	Inadequate consideration of health protocols by psychiatric patients
		Lack of facilities and personal protection equipment (PPE)
	Failure to complete the treatment process for patients	Early discharge of patients due to Corona disease
		Transferring patients to COVID-19 treatment centers
	Institutional Policies	limitation in visiting psychiatric patients
		Caring for positive COVID-19 patients in non-standard conditions
		Enforcing the staff to work overtime due to lack of human force
		Early return of nursing staff from Corona leaves due to lack of human force

### Emotional responses

These emotions are attributed to traits in nurses which made them unable to perform their moral duties during the COVID-19 pandemic. Fear and doubt of nurses arising from the unknown aspects of the disease was a main source of moral distress.

### Fear

Fear of COVID-19 disease and ineffective care of the patients by the nurses caused them moral distress. Participant No. 2 stated in this regard:

“I’m terrified of approaching patients. Patients who do not wear masks. We also communicate with them to talk, give medication, and many other things … Our care quality is deficient…We have no choice …we are forced for the sake of our health and our family … But the patient also suffers… I felt moral distress about this issue….”

Psychiatric nurses also reported this fear in physicians and stated that they avoided patients and did not visit them in their beds. Participant No. 3 said:

“Since this disease (COVID-19) has got spread, the doctor comes and visits all the patients in 10 min and leaves the ward. He is afraid to talk to the patient. He ignores what we say. Whatever I say, the patient does not sleep well at all; he’s talkative and aggressive. For example, he says to continue the previous treatment! He is not even willing to visit the patient for a few minutes. Well, all this upsets me. I say, what happens to the patients?! This is not moral at all.it is not legal … this makes me morally distressed…”

### Doubt

Due to the special conditions of psychiatric patients and disregarding health protocols these patients, are more likely to have “doubts” about their work than other nurses who work with patients who follow the protocols. Participant number four said in this regard:

“I often decided to quit my job because of the dangers of working with psychiatric patients in this situation (Corona), but every time I told myself I chose this job, and I should not work only in good conditions. I’m accountable to my countrymen. I am a nurse. If I am not, who will take care of these patients?! This is not fair. But this doubt still exists. There is left, at least, for the health of my family … I feel moral distress…”.

### Relational factors

In this category, psychiatric nurses pointed to challenges that the COVID pandemic has had on interpersonal relationships and stated that this critical situation has had an impact on the relationships between nurses, the relationships between nurses, physicians and patients, and has caused moral challenges, Failure to act according to professional values and create moral distress.

### Nurses’ relationship with each other

Nurses have talked about the problems resulting from healthcare changes during the COVID-19 outbreak in nurses’ interpersonal relationships. In the category of “nurses’ relationship with each other,” many nurses stated that they witnessed colleagues’ negligence in doing their responsibility of caring for the patients due to their fear of COVID-19 disease. In this regard, participant No. 3 said:

“Some colleagues do not do their duties. For example, I saw that he did not give his patient medicine or check his blood sugar. I know that the patient has a psychiatric problem. But he does not have a serious physical illness. So, there is no reason not to care for him just for fear of Corona. I see these things and cannot say anything to my colleague. Then, I suffer from guilt feeling and moral distress. I tell myself I should not allow my colleague to miss the patient”.

### Nurse-physician relationship

In the “nurse-physicians relationship” category, nurses have pointed out that physicians ignore nurses’ viewpoints in making therapeutic decisions. Participant No. 8 stated:

“The patient still has hallucinations. He has potential suicidal and homicidal thoughts, but because he is suspected of having COVID-19 disease, the physician didn’t visit him at all. He just wrote the order for transferring the patient. He is not even waiting for the PCR test result. Well, these things make me upset and morally distressed. Why ignore nurses’ opinions in decision-making by physicians? We are in close contact with the patient, so we understand it better “.

### Relationship with patients

In the category of “relationship with patients,” psychiatric nurses stated that they have to take distance from patients due to the outbreak of this disease. A psychiatric nurse's first and most important task is to establish therapeutic communication with the patient, which requires face-to-face contact. Participant number two said in this regard:

“I’m upset that my patients do not take the protocols, and I have to keep my distance. I always talk to my patients a lot. I listened to them, but now, because of this disease, I’ve missed my close contact with patients “… Sometimes I tell myself I have no right to cut ties with my patient for fear of Corona”.

The nurses also complained about the inadequate communication between physicians and patients so that physicians do not answer patients’ questions since the outbreak of Corona disease. Furthermore, the therapy process has been disrupted due to inadequate medical examinations by physicians.

### Institutional factors

In this category, psychiatric nurses stated the reasons for the inability to act ethically due to uncontrollable environmental conditions due to COVID disease and emergence of moral distress.

### Lack of attention to health instructions

“Lack of attention to health instructions” is one of the conditions that existed in psychiatric hospitals due to patients’ unique needs, their poor insight into their disease, and, sometimes, having delusions and hallucinations. Thus, they do not follow health guidelines such as wearing masks or social distance, which has caused nurses and physicians to be concerned about COVID-19 disease. Participant number 6 said:

“Patients do not wear masks at all while taking medication, or they sit in groups together without observing the distance without the mask. If one of them is a carrier, they all get Corona and transmit it to the nurses. So, I keep away from the patients as much as possible, not for myself, but for my family. “I know this is not a moral behavior, but I have no choice.”

### Failure to complete the treatment process for patients

Psychiatric nurses also complained about the insufficient personal protective sets of equipment such as gloves and masks, especially in the early stages of the disease. In this regard, participant number 5 said:

“Why don’t the managers understand that psychiatric nursing patient in this situation is much more complex than caring for other patients. Why should the masks and personal protective equipment quota be half of the other hospitals in our hospital? Fair? “No, it is not moral. Well, this has a direct effect on patient care. The quality of the nurse’s care will decrease, and then I feel moral distress ….”

### Institutional policies

In this area, we narrate the policies made by higher decision-makers that the nurses are forced to implement. This area includes “organizational policies.” In this category, nurses stated that patients could visit their relatives limitedly due to prevent the spread of COVID-19 disease. In this regard, participant No. 4 has stated:

“I can see the suffering and restlessness of the patients. Here is a prison for the patient. Meeting family is forbidden, the ward’s phone is mostly broken, and they also do not have mobile phones. Well, the patients become depressed. They are all dissatisfied and say it wishes we got Corona but let to visit our family. These issues upset me as a nurse, and they cause me moral distress.”

Also, patients who had COVID-19 disease but were in an excellent general condition remain in the wards, based on physicians’ decisions, without health protocols and isolated. In this regard, participant No. 5 said:

“The doctor does not want to visit the patient. We take care of the patient day in and day out. Why don’t they isolate the patients?? It is not ethical. Why don’t the university officials allocate a single psychiatric ward for these patients? These issues cause moral distress among the nurses because there is nothing she can do.”

Participant No.10 said: “Decrease of sick leave time hurts the system: Well, my colleague is still a carrier; he goes back to work, he infects a few other nurses again.”

They were also dissatisfied with the rules and regulations laid down by managers in Corona’s pandemic conditions. They stated that they had to work overtime due to their co-workers’ illness and return early from Corona sick leave due to a lack of nursing staff.

## Discussion

This qualitative study identified factors that evoke moral distress among Iranian psychiatric nurses during the COVID-19 pandemic.

In the category of emotional responses, psychiatric nurses have repeatedly expressed that they experienced severe concern about being exposed to the virus during care provision, being doubtful to quit the nursing profession and working in an unsafe condition, or going on nursing where their lives are in danger. So, they could not fulfill their moral and professional duties in the COVID-19 pandemic and felt that they had no power to change the situation. The nurses’ commitment to giving care and the right and responsibility to protect themselves and their families created a conflict between a psychiatric nurse's professional duty and personal duties.

Hossain and Clatty examined 2020 nurses’ self-care strategies during the COVID-19 pandemic and noted this conflict among nurses. They stated that nurses, like other people, have families and loved ones in their lives. They find themselves in competition with commitments to work, family, and loved ones and conflict between professional duty and responsibility to their families [20].

Zhang et al. [21] in China showed controversy between fear of being infected with COVID-19 () and their professional commitment in nurses. The feeling of powerlessness or inability to perform the moral action perceived by nurses has been identified as one of the leading causes of moral distress. This is usually due to nurses’ personal feelings, such as doubt and fear [22].

Also, observing physician’s fear of contracting the COVID disease and psychiatrists' avoidance of patients caused moral distress in psychiatric nurses. In this regard Abdelghani et al. [23] reported There was a robust correlation between fears and higher burnout symptoms, and poor quality of life among physicians.

Empowering nurses to deal with their doubts and fears can reduce moral distress and also, ethics education that emphasized moral debriefing and decision-making is recommended.

In the relational factors, psychiatric nurses narrated the changes during the pandemic in the relationships between them and their colleagues, physicians, and patients. Working with nurses who are negligent in providing care to patients due to fear of being exposed to the virus has caused them moral distress. Silverman also pointed out that nurses who do not provide proper care to patients have driven them into moral distress [24]. Empowering nurses to reduce their negligence in performing their duties, especially in critical situations, is recommended. This can prevent feelings of self-blaming, powerlessness, and moral distress in nurses.

Nurses talked about the challenges they encountered, especially physicians, in dealing with patients with suspected or developing COVID-19 disease. Keeping distance from psychiatric patients and visiting them with high restrictions by physicians due to their fear of Corona disease is an example of not performing the professional and moral duties of the physicians that are considered unethical by nurses. On the other side, given that Nurses do not have the ability and power to object to physicians, they are not allowed to share in therapeutic decisions made by physicians.

Providing an environment in which the nurse can participate in clinical decisions and solve ethical issues and express their views without fear of organizational and occupational issues is very effective in reducing moral distress, which is necessary in this regard. Managers should provide the necessary support to nurses.

In a study conducted by Silverman et al. [24] about working in COVID-19 wards, one causes of moral distress in nurses was the observation of patients suffering due to improper performance of physicians in the management of their treatment. This result is compatible with the results of our study.

The lack of proper communication between nurses and their patients due to fear of COVID-19 disease was also reported in this study. Other studies conducted among nurses in COVID-19 conditions have also shown that nurses and patients must maintain a certain distance when communicating to reduce the prevalence of infection. This leads to insecure feelings in patients and nurses so they feel unable to support their patients [25–27]. Since the ethical climate of any organization has a significant effect on the level of moral distress [28, 29], creating a supportive climate with an emphasis on strengthening communication skills in nurses besides, physicians can be effective in enforcing the ethical climate and reducing moral distress among nurses.

In the institutional factors category, the issue, which is specific to psychiatric hospitals and their patients, which has caused moral distress in nurses, is disregarding protective guidelines by patients. Nurses working in non-psychiatric hospitals care for patients who have enough insight into their illness and adherence to protective guidelines. But, psychiatric nurses care for patients who do not only care about their condition but also do not adhere to the protective guidelines, such as wearing masks and observing social distance. They also do not have insight and are inconsiderate of nurses’ education. So, this issue has caused them to be more worried about Corona disease than other nurses.

All these circumstances caused the nurses to avoid the patients and feel moral distress by avoiding their patients. This issue, along with the lack of facilities and the limited access to personal protective equipment in psychiatric hospitals, has also intensified the moral distress of psychiatric nurses. Injustice and discrimination have been reported in studies on patient triage and the allocation of critical beds and ventilators during pandemics [26, 30]. A survey conducted by Kalateh Sadati among 24 Iranian nurses in 2020 examined their experiences during the COVID-19 outbreak; they found that Iranian nurses were also in critical condition during the attack. Lack of personal protective equipment, uncertainty about the treatments provided to their patients, and the need for psychosocial support is widespread among all nurses [31]. Still, this discrimination mentioned by psychiatric nurses is uniquely seen in psychiatric hospitals.

Like other nurses in this pandemic, psychiatric nurses have been affected by the critical situation. However, the impacts of this crisis on this group of nurses have been ignored, unfortunately, due to the focus of managers on the COVID-19 central hospitals [32]; These managers and policymakers should be paying more attention to the needs of psychiatric hospitals by allocating sufficient and appropriate budget and facilities.

The implementation of some institutional policies, such as visit limitation of psychiatric patients by their families, was the cause of moral distress. The critical role of the family in the treatment of psychiatric patients is evident, so one of the supplementary treatments for these patients, along with medication, is family therapy. Also, studies have shown the increased risk of loneliness, confusion, and delirium due to a lack of family visits during the COVID-19 outbreak [25, 26]. Observation of patients’ suffering and restlessness by psychiatric nurses has caused moral distress.

The managers seem to be necessary to promote these policies to improve the quality of care, increase patient satisfaction, reduce moral distress in nurses, and make it possible that the patients can visit their families in safe conditions based on health-protective instructions.

Another institutional policy referred to psychiatric nurses was obligatory overtime work and early return from sick leave. In the critical situation, along with the shortage of nursing staff in the Iranian health care system, some managers have been forced to make tough decisions to compensate for the lack of human forces. The study of Navab et al. [33] examined patient care during the COVID-19 pandemic, pointing to the shortage of human passion in critical situations, which has reduced the quality of care and unsatisfied nurses ‘sense of support and has prevented from taking nurses’ duties.

As the main strength of this study, we conducted one of the qualitative studies that explored situations and factors that caused moral distress based on experiences of the psychiatric nurses who care for COVID-19 psychiatric patients. This study limitations includes the fact that this study was conducted during the COVID-19 Pandemic and nurses were under high work pressure, it was challenging to coordinate interview sessions with them and face-to-face interviews due to the need to observe social distance and health guidelines. Also, another limitation is small sample size and convenience sampling due this situation therefore, the participants may not be representative of a general population of Iranian psychiatric nurses. Although most conditions in hospitals in this crisis were almost the same as in other hospitals, the results of this study cannot be generalizable to other hospitals which experienced different degrees of COVID-19 concern.

## Conclusion

This study showed that psychiatric nurses paid attention to the ethical dimensions of care in psychiatric patients during the COVID-19 pandemic. New dimensions of the factors causing moral distress in nurses have been discovered in COVID-19 pandemics, some of which were specific to psychiatric nurses.

The variation in the causes of moral distress among psychiatric nurses and other nurses was due to the following factors: nature of the disease of psychiatric patients, lack of facilities in psychiatric hospitals such as inadequate isolation rooms, care facilities for positive patients, as well as insufficient attention of managers and policymakers to these hospitals, the negligence of psychiatric nurses in allocating facilities and personal protection equipment and facilities provided for other nurses in these conditions such as employment factors and benefits and material and spiritual incentives.

The present study’s findings suggest that the managers and policy-makers must decrease the irreversible effects of moral distress by allocating sufficient facilities and personal protection equipment to psychiatric hospitals. Also, because ethical care occurs in intra-professional and inter-professional communication [24], it is necessary to improve communication skills by strengthening the moral climate to create a contributing atmosphere to control the crisis.

It is suggested that future studies address the strategies of psychiatric nurses and strengths and weaknesses of the healthcare system in dealing with moral distress in COVID-19 crisis based on our finding.

## Supplementary Information

**Additional file 1**. Interview Guide.

## Data Availability

The datasets used and analyzed during the current study are available from the corresponding author on reasonable request.
