# Supplementary material for: Moral distress in psychiatric nurses in Covid-19 crisis
Source: BMC Psychol. 2023 Feb 17;11:47. doi: 10.1186/s40359-023-01048-y (PMC9936116; doi:10.1186/s40359-023-01048-y)
Supplement: Supplementary file 1 — Additional file 1. Interview Guide. [file 40359_2023_1048_MOESM1_ESM.docx]

**Interview Guide**

Thank you for participating in the present study. The objective of the study is to determine the factors that caused moral distress to psychiatric nurses who face and care for COVID-19 patients. You are going to be asked a few questions about the subject of the study. If you find a question ambiguous, please ask us for clarification. All the information collected during the interview will be confidential and only the research team will have access to it. The interview will be audio recorded to ensure that the researchers will not miss any significant points. The recorded interview and your personal information will be treated as confidential. Your interview will be assigned a code. Do you consent to the recording of your interview with a voice recorder?

**Personal information**

Name:

Gender:

Marital status:

Professional position:

Work experience:

Educational background (highest degree):

Managerial backgrounds:

Time employed:

**General question**

Can you describe your experiences of a work shift in which you faced or cared for a COVID19 patient?

**Specific questions**

What are your experiences about the ethical issues when you faced and cared for COVID-19 patients?

What factors causes moral distress when face and care for COVID-19 patients?
